# L-Ornithine L-Aspartate for the Treatment of Sarcopenia in Chronic Liver Disease: The Taming of a Vicious Cycle

**DOI:** 10.1155/2019/8182195

**Published:** 2019-04-28

**Authors:** Roger F. Butterworth

**Affiliations:** Department of Medicine, University of Montreal, Canada

## Abstract

Sarcopenia is a common complication of cirrhosis with a negative impact on posttransplant outcome, health-related quality of life (HRQOL), and patient survival. Studies in experimental animals and in patients demonstrate that ammonia is directly implicated in the pathogenesis of sarcopenia in cirrhosis via mechanisms involving increased expression of myostatin and of autophagy markers such as LC3 lipidation and p62 leading to muscle dysmetabolism and sarcopenia. Paradoxically, skeletal muscle replaces liver as the primary ammonia-detoxifying site as a result of the modification of genes coding for key proteins implicated in ammonia-lowering pathways in cirrhosis. Thus, a vicious cycle occurs whereby hyperammonemia causes severe muscle damage and sarcopenia that, in turn, limits the capacity of muscle to remove excess blood-borne ammonia and the cycle continues. Randomized clinical trials and meta-analyses confirm that L-ornithine L-aspartate (LOLA) is an effective ammonia-lowering agent currently employed for the treatment of hepatic encephalopathy (HE) that stimulates both urea synthesis by residual hepatocytes and muscle glutamine synthesis together with putative hepatoprotective actions. Treatment of cirrhotic patients with LOLA limits ammonia-induced sarcopenia by improving muscle protein synthesis and function. It is conceivable that the antisarcopenic action of LOLA contributes to its efficacy for the treatment of HE in cirrhosis.

## 1. Introduction

Sarcopenia or severe muscle wasting is a common complication of chronic liver diseases including nonalcoholic fatty liver disease (NAFLD) [1, 2] as well as cirrhosis [3, 4] where it is associated with a poor prognosis with negative impact on survival, health-related quality of life, and posttransplant outcomes. Median 6-month survival rates for sarcopenic patients with cirrhosis are significantly lower compared to nonsarcopenic patients.

The presence of sarcopenia is a predictor of a spectrum of central nervous system(CNS) complications of cirrhosis that include hepatic encephalopathy (HE) ranging in severity from minimal (or covert) to higher grades characterized by stupor and coma [5] as well as HE associated with the transjugular intrahepatic stent shunt(TIPS) procedure [6].

The present article is a review of evidence in support of the twin hypotheses that (1) hyperammonemia initiates a vicious cycle whereby reduced muscle protein synthesis and autophagy characteristic of sarcopenia reduces the capacity of muscle to remove blood-borne ammonia leading to exacerbation of hyperammonemia and (2) treatment with the ammonia-lowering agent L-ornithine L-aspartate (LOLA) has the potential to break this cycle.

## 2. Sarcopenia in Chronic Liver Disease: The Link to Hyperammonemia

Excess quantities of ammonia are, under normal physiological conditions, removed almost exclusively by the liver either via urea synthesis by periportal hepatocytes or by conversion to glutamine by the enzyme glutamine synthetase (GS) located in perivenous hepatocytes. These processes occur simultaneously. Results of neuropathologic, spectroscopic and imaging investigations continue to support the hypothesis that increased concentrations of ammonia play a major role in the pathogenesis of the CNS complications of cirrhosis [7]. In chronic liver diseases, this hepatic ammonia-trafficking pattern undergoes modification since the capacity for both urea synthesis and glutamine production is seriously impaired.

Under these circumstances, skeletal muscle takes on an important role for ammonia removal and, unlike the liver, this process occurs exclusively via glutamine synthesis since muscle cells do not express the constituent enzymes of the urea cycle. Evidence for this metabolic switch from liver to muscle is predicated on the results of studies in patients with chronic liver failure. In the first such study, arteriovenous differences for ammonia and glutamine were measured across the forearm in 14 patients with decompensated cirrhosis and hyperammonemia. Fractional extraction of ammonia was significantly decreased in 5 patients with gross muscle wasting [8] and arteriovenous for glutamine in the patient group was increased several fold suggesting that muscle plays a key role for the disposal of ammonia in patients with cirrhosis and that a major fraction of ammonia taken up by muscle is released as glutamine. These findings were confirmed in a subsequent study of the dynamics of ammonia metabolism in patients with chronic liver disease using ^13^NH_3_ where significant metabolic trapping of ammonia by muscle was observed [9].

**Figure 1:**
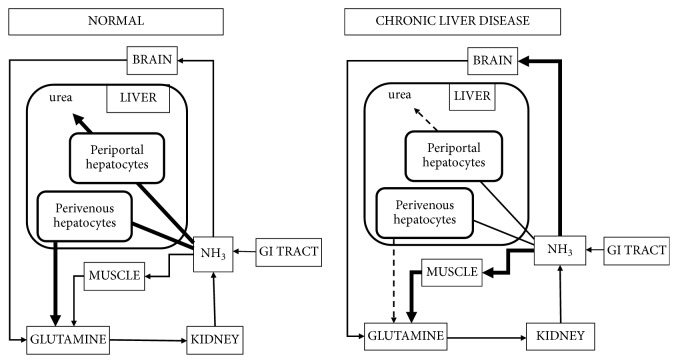
Classical view of interorgan trafficking of ammonia (derived principally from protein digestion in the gastrointestinal tract) between liver, muscle, and brain under normal physiological conditions compared to chronic liver disease. Note the decrease of ammonia removal as urea and glutamine by periportal and perivenous hepatocytes, respectively, in chronic liver disease accompanied by increased removal of ammonia by skeletal muscle and increased uptake of ammonia by brain, a key factor in the pathogenesis of HE.

Molecular studies in experimental animals suggest that this alternative pathway for ammonia removal implicating skeletal muscle in chronic liver diseases is facilitated by posttranslational induction of GS [10] together with increases in expression of ammonia transporters [11]. The metabolic transformations implicated in the alternate pathway are depicted in a simplified schematic manner in Figure 1.

Given the evidence that muscle becomes the major pathway for ammonia removal in chronic liver disease, it is evident that sarcopenia would likely result in worsening of hyperammonemia. Such is observed in the studies described above [8, 9, 12].

Results of recent* in vitro* and animal model investigations suggest that ammonia* per se* is implicated in the pathogenesis of sarcopenia in chronic liver diseases. In a series of ground-breaking studies, exposure of differentiated myotubes to millimolar concentrations of ammonia for 24 h resulted in decreased myotube diameters, decreased protein synthesis, and increased expression of a range of markers of autophagy [12]. The investigators then went on to demonstrate that removal of the ammonia resulted in significant attenuation and, in some cases, complete reversal of these changes.

Making use of the well-established end-to-side portacaval-shunted (PCA) rat preparation recommended by The International Society for Hepatic Encephalopathy and Nitrogen metabolism (ISHEN) for the study of mechanisms implicated in the pathogenesis of chronic liver diseases and their complications [13], Dasarathy and colleagues continued to address the role of hyperammonemia in the pathogenesis of sarcopenia in chronic liver disease [12]. One week after PCA, lean body mass, grip strength, muscle mass, and muscle fibre diameter were significantly reduced compared to pair-fed control animals and these changes occurred in parallel with significant increases of both blood and muscle ammonia concentrations.

The findings of hyperammonemia-mediated autophagy in skeletal muscle were subsequently confirmed in patient material leading to the suggestion that such changes contribute to sarcopenia in cirrhosis [14].

**Figure 2:**
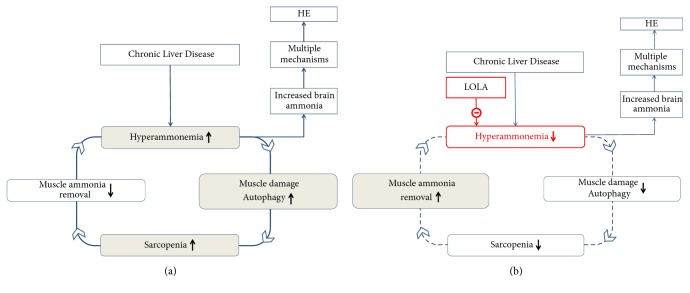
(a) Schematic representation of a vicious cycle by which hyperammonemia in cirrhosis resulting from diminished hepatic ammonia removal and portal-systemic shunting of venous blood results in muscle damage characterized by impairment of proteostasis and autophagy, the hallmarks of sarcopenia. Sarcopenia results in decreased capacity for ammonia removal by the muscle leading to enhancement of hyperammonemia and the cycle continues. (b) Treatment with LOLA results in reduction of hyperammonemia via metabolic and hepatoprotective mechanisms leading to a reduction of muscle damage and sarcopenia with consequent restoration of muscle glutamine synthesis, an additional mechanism implicated in the lowering of blood ammonia by LOLA in chronic liver disease. Direction and magnitude of changes in components of the cycle in this figure are indicated by arrows.

Based upon the above findings, it appears that a vicious cycle occurs in chronic liver disease whereby hyperammonemia resulting from reduced hepatic removal of ammonia in the form of urea and/or glutamine causes severe muscle dysmetabolism and autophagy that are characteristic of sarcopenia. The resulting impairment of muscle function and loss of the alternative pathway for ammonia removal by muscle in chronic liver disease leads to exacerbation of hyperammonemia and the cycle continues (Figures 2(a) and 2(b)).

The presence of such a cycle provides a plausible explanation for the well-established clinical observation that patients with cirrhosis and severe muscle loss fare poorly. In a study of an unselected series of patients with cirrhosis who had emergency portacaval shunt surgery, severe muscle wasting was associated with a worse prognosis for survival [9] with a significant negative impact also upon quality of life, posttransplant outcomes, and an increased prevalence of extrahepatic complications including HE. The effective lowering of blood ammonia may therefore be of key importance for the effective management of sarcopenia in cirrhosis [15].

## 3. LOLA and Sarcopenia in Cirrhosis

LOLA is used routinely in the clinic for the effective reduction of circulating ammonia in patients with cirrhosis and this was confirmed in a recent systematic review and meta-analysis [16]. LOLA had previously been shown to lower blood ammonia in rats made hyperammonemic by PCA [17], i.e., the same animal model used to demonstrate the effects of hyperammonemia on skeletal muscle integrity [12].Treatment of PCA rats with LOLA and addition of the antibiotic rifaximin resulted in significant improvements in lean body mass, grip strength, skeletal muscle mass, and muscle fibre diameters as a function of marked reductions in circulating and skeletal muscle ammonia concentrations [12]. Protein synthesis rate in gastrocnemius muscle that had been significantly reduced following PCA was improved by the ammonia-lowering strategy.

These findings add to an emerging body of evidence suggesting a role for LOLA in the treatment of sarcopenia in cirrhosis. For example, in a metabolic study of 16 patients with cirrhosis and sarcopenia randomized to receive LOLA or placebo, muscle protein synthesis rates measured in percutaneous biopsies of* anterior tibialis* muscle, improved significantly in the LOLA treatment group [18]. The findings from this study also demonstrated improvement of muscle protein synthesis in response to feeding following LOLA treatment.

## 4. Mechanisms

### 4.1. Mechanisms Related to the Effects of Ammonia on Muscle Leading to Sarcopenia

Hyperammonemia leads to the transcriptional upregulation of myostatin, a TGF*β* super-family member [19], and studies in differentiated murine myotubes exposed to ammonia or in muscle from hyperammonemic PCA rats, myostatin expression was likewise increased. Results of these studies also revealed impaired signalling of mammalian target of rapamycin complex-1 (mTORC1) and increased phosphorylation of eukaryotic initiation factor 2-alpha (eIF2*α*), a translational repressor [20] having the potential to result in reduced muscle protein synthesis [12]. Hyperammonemia also led to increased expression of autophagy markers such as LC3 lipidation and p62 expression in protein extracts [12]. Ammonia lowering resulted in significant attenuation of the upregulation of myostatin expression and the associated decreased muscle protein synthesis in both the* in vitro* and* in vivo* experiments and, additionally, led to reversal of the elevated markers of autophagy [14].

### 4.2. Mechanisms Related to the Beneficial Effects of LOLA on Sarcopenia in Cirrhosis

Studies in both experimental animal models of HE [17] as well as in patients with cirrhosis and hyperammonemia [21] have consistently shown that LOLA is effective for the lowering of circulating blood ammonia resulting in an improvement in HE severity. The mechanism responsible involves both liver and skeletal muscle. Since L-ornithine is a key urea cycle intermediate, it has the capacity to stimulate the conversion of ammonia to urea by residual periportal hepatocytes [17]. Simultaneously, ammonia is also removed to a large extent via increased conversion to glutamine principally by the muscle [9, 17] via GS since transamination of L-ornithine provides glutamate, the obligate substrate for GS. Via these two independent mechanisms (urea synthesis in the liver and glutamine synthesis in muscle), LOLA treatment lowers both blood and muscle ammonia resulting in improved skeletal muscle phenotype and function together with attenuation of the deleterious molecular perturbations caused by ammonia [12].

However, it is conceivable that mechanisms other than (or in addition to) the simple ammonia-lowering action of LOLA are implicated in the beneficial effects of LOLA with respect to sarcopenia in cirrhosis. For example, there is increasing evidence to suggest that LOLA has hepatoprotective properties in patients with cirrhosis [22]. Evidence is multidimensional and is based upon the results of clinical trials of LOLA in which improvements in circulating liver transaminases and bilirubin as well as prothrombin times occur [22–25]. Improvements of Child Pugh and MELD scores have also been reported in LOLA-treated patients with chronic liver disease where improvements in liver function were accompanied by significant reductions in circulating ammonia as well as improvements in cognitive function [23].

Putative mechanisms proposed to explain the hepatoprotective properties of LOLA include antioxidant properties mediated via increased synthesis of glutathione [26] derived from the transamination of L-ornithine via glutamate as well as improvements in hepatic microcirculation resulting from increased synthesis of nitric oxide (NO) [27] resulting from increased synthesis of L-arginine [21], the obligate substrate for nitric oxide synthase. While further studies are required to determine if NO is implicated in the pathogenesis of sarcopenia in cirrhosis, it is interesting to note that increased NO production in muscle leading to the S-nitrosylation of calpain results in slowing of sarcopenia in aging [28]. Whether or not a similar mechanism occurs in cirrhosis-related sarcopenia awaits the results of ongoing investigations.

## 5. Conclusions

Interorgan trafficking of ammonia is modified in chronic liver disease whereby the normal removal of ammonia by the liver as urea or glutamine gives way to its incorporation into glutamine by skeletal muscle. This metabolic adaptation results from the posttranslational induction of muscle GS together with increased expression of the ammonia transporter in muscle. Paradoxically, ammonia has been shown to have deleterious effects on muscle protein synthesis and results in autophagy. These two processes, together, constitute sarcopenia.

A vicious cycle is created whereby hyperammonemia causes muscle damage (sarcopenia) that limits the capacity of muscle to fulfill its alternative ammonia-lowering role in chronic liver disease. Evidence for the existence of the cycle involving the liver-muscle axis is predicated on the results of experiments using* in vitro* techniques as well as in preclinical and preliminary clinical investigations in patients with cirrhosis.

LOLA has the capacity to restore muscle protein synthesis in response to feeding in patients with cirrhosis, a finding that was recently confirmed. The mechanism by which LOLA is beneficial for the treatment of sarcopenia in cirrhosis relates primarily to its ammonia-lowering action. In addition, LOLA has hepatoprotective properties mediated via the transformation of L-ornithine into key substrates including glutamine, glutathione (an antioxidant), and L-arginine, the substrate for nitric oxide synthase, the enzyme responsible for NO production.

Finally, the focus of the present review has principally been directed at the potential benefits of LOLA treatment for sarcopenia in cirrhosis. However, there is emerging evidence to support the notion that it may also be beneficial in patients with sarcopenia related to NAFLD/NASH [29]. For example, in a study of 463 patients with fatty liver, 29% of whom were nonalcoholic, treated with oral LOLA for up to 60 days, significant decreases of liver enzymes [30] were noted and in a subsequent RCT, 72 patients treated with oral LOLA for 12 weeks manifested significant dose-related reductions of transaminases together with decreases in triglycerides and improvements in liver/spleen ratios [31]. A preliminary report described improvements in hepatic microcirculation in patients with NASH treated with LOLA [32].

Confirmation of these initial findings of a beneficial effect of LOLA for the treatment of sarcopenia in chronic liver diseases (both NAFLD and cirrhosis) is now required in adequately powered, well-controlled trials in order to demonstrate that LOLA monotherapy provides an effective agent for the prevention and treatment of sarcopenia in these chronic liver diseases.
